# Efficacy and safety of low-power (20 W) catheter ablation for typical atrioventricular nodal reentrant tachycardia: a prospective multicenter study

**DOI:** 10.3389/fcvm.2026.1949109

**Published:** 2026-10-30

**Authors:** Lae-Young Jung, Chang Hee Kwon, In Geol Song, Ji Hyun Lee, Minsu Kim

**Affiliations:** 1 Division of Cardiology, Jeonbuk National University Hospital and Jeonbuk National University Medical School, Jeonju, Republic of Korea; 2 Division of Cardiology, Department of Internal Medicine, Konkuk University Medical Center, Konkuk University School of Medicine, Seoul, Republic of Korea; 3 Department of Cardiology, Konyang University Hospital, Daejeon, Republic of Korea; 4 Cardiovascular Center, Seoul National University Bundang Hospital, Seongnam, Republic of Korea; 5 Department of Cardiology in Internal Medicine, School of Medicine, Chungnam National University, Chungnam National University Sejong Hospital, Sejong, Republic of Korea

**Keywords:** atrioventricular nodal reentrant tachycardia, low-power ablation, prospective multicenter clinical trials, radiofrequency catheter ablation, treatment outcome

## Abstract

**Background:**

Atrioventricular nodal reentrant tachycardia (AVNRT) is the most common supraventricular tachycardia. Although catheter ablation is the definitive treatment, atrioventricular (AV) block requiring permanent pacemaker implantation remains a major safety concern.

**Objective:**

To evaluate the efficacy and safety of a low-power (20 W) slow pathway ablation strategy for typical AVNRT.

**Methods:**

In this prospective multicenter study, 203 consecutive patients (mean age 54.4 ± 17.3 years; 59.6% women) underwent slow pathway ablation initiated at 20 W, with power escalation as needed. Variables associated with requiring higher power were analyzed using univariable and multivariable logistic regression.

**Results:**

Initial success at 20 W was achieved in 170 patients (83.7%); 33 patients (16.3%) required higher power, with an overall acute success rate of 99.5%. Compared with patients who achieved success at 20 W, those requiring higher power were younger and had higher pre-ablation heart rates. On univariable analysis, younger age [odds ratio [OR] 0.66 per 10-year increase, 95% confidence interval [CI] 0.54–0.83; *p* < 0.001] and higher pre-ablation heart rate (OR 1.88 per 10-bpm increase, 95% CI 1.30–2.73; *p* < 0.001) were associated with the need for higher power. Transient AV block occurred in 1.9% of patients, and no cases of permanent AV block (0%, 95% CI 0%–1.8%) or pacemaker implantation were observed. During 18.3 ± 4.1 months of follow-up, recurrence occurred in two patients (0.9%).

**Conclusion:**

An initial 20 W ablation strategy provides high efficacy with a favorable safety profile and no permanent AV block observed in this cohort. Younger age and a higher baseline heart rate predicted the need for power escalation, supporting an initial low-power approach for AVNRT ablation.

## Introduction

Slow pathway modification using radiofrequency (RF) energy is the gold standard for treating atrioventricular nodal reentrant tachycardia (AVNRT) due to its high success rates (1). The primary complication of AVNRT ablation is complete atrioventricular (AV) block, with reported incidences ranging from 0.1% to 0.8% (2–4). Additionally, late AV block requiring permanent pacemaker implantation (>1 month after ablation) has been reported in 0.4% of patients (5). Despite the clinically meaningful risk of acute and late AV block, data remain limited regarding optimal RF power settings for AVNRT ablation.

Slow pathway modification is generally performed by identifying the anatomical target site, confirming electrogram characteristics, and initiating ablation. Although this approach is widely practiced, no standardized protocol exists for optimal energy delivery or endpoint definitions, resulting in substantial procedural variability among operators. Prior studies have reported a broad range of power settings, from 20 W to 50 W, with some adopting stepwise power-escalation strategies (6–8).

Notably, Bortone et al. reported that slow-accelerated junctional rhythm occurred at a mean power of 18.5 W during slow pathway modification. Furthermore, a recent study by Kawaji et al., using a near-field detection algorithm–guided approach, suggested that the slow pathway is relatively superficial, supporting the feasibility of effective ablation using lower energy levels (9). However, evidence for low-energy ablation remains limited, with most studies conducted in single center settings and involving small, highly selected patient cohorts (8, 10).

The present study was designed to evaluate the feasibility, efficacy, and safety of slow pathway ablation initiated at 20 W with selective power escalation in patients with typical AVNRT. We hypothesized that this low-power-first strategy would achieve a high acute procedural success rate while limiting procedure-related AV block.

## Methods

### Study patients

We conducted a prospective multicenter trial at three tertiary referral hospitals—Jeonbuk National University Hospital, Konkuk University Medical Center, and Chungnam National University Sejong Hospital—between May 2017 and February 2022. A total of 203 patients undergoing slow pathway ablation for typical AVNRT with 20 W energy were enrolled: 48 patients at Jeonbuk National University Hospital, 56 at Konkuk University Medical Center, and 99 at Chungnam National University Sejong Hospital. All patients provided informed consent before the electrophysiology study and catheter ablation procedure. The study protocol was reviewed and approved by the institutional review board of each participating hospital.

### Definition

Typical AVNRT was deﬁned by an atrial-His/His-atrial ratio >1 and a His-atrial interval ≤70 msec (11). The echo window was defined as the interval during programmed stimulation with 10 msec decrements, in which a single echo was observed after ablation. Transient AV block was defined as second-degree AV block (Mobitz type I or II) or third-degree AV block that resolved completely during the procedure. Recurrence was defined as documented supraventricular tachycardia on a 12-lead ECG, 24-hour Holter monitoring, portable ECG devices, or repeat electrophysiology study.

### Electrophysiology study and ablation

RF energy was delivered in pulses of up to 60 s at optimal sites, with a maximum temperature of 60 °C (12). Electrophysiology studies were performed in the fasting state. All patients discontinued antiarrhythmic medications for at least 5 half-lives. Conscious sedation with midazolam, propofol, and fentanyl was administered. Venous access was obtained through both groins, and multipolar catheters were placed in standard positions: the high right atrium, coronary sinus, right ventricular apex, and His bundle. Sustained typical AVNRT was induced in all patients using programmed atrial and ventricular stimulation at different cycle lengths, with isoproterenol administered when necessary.

Slow pathway ablation followed standard technique using three types of conventional 4-mm ablation catheters (Blazer®, Boston Scientific, Natick, MA, USA; EZ Steer®, Biosense Webster Inc. Irvine, CA, USA; Safire®, St. Jude Medical, Minnetonka, MN, USA). All procedures were performed by three experienced electrophysiologists, one at each participating center, each of whom performed more than 50 AVNRT ablation procedures annually and had performed more than 500 AVNRT ablation procedures in total. Catheter selection among these three models was based on the treating operator's preference. As contact force-sensing catheters were not available, an impedance drop of ≥10% was used as a surrogate marker of adequate catheter-tissue contact and was targeted during ablation. The ablation catheter was positioned in the posterior part of the atrial septum, between the tricuspid annulus and the coronary sinus ostium, until the atrial-to-ventricular electrogram ratio ranged from approximately 1: 10–1:3, with a delay in the atrial electrogram relative to the His bundle recording. When multicomponent or low-amplitude high frequent potentials were identified, RF energy was applied for up to 20 s until a junctional rhythm with 1:1 retrograde ventriculoatrial (VA) conduction was observed (13, 14). Slow-accelerated junctional rhythm—defined as ≥5 junctional beats with a mean cycle length >350 msec (15)—was considered indicative of effective modification, after which RF energy was continued for up to 60 s. If fast-accelerated junctional rhythm (≥5 junctional beats with a mean cycle length ≤350 msec) or VA block occurred (16), RF delivery was immediately discontinued, and the catheter was repositioned. Maximum temperature was defined as the highest temperature recorded exclusively during the initial 20 W ablation phase, before any power escalation. If slow-accelerated junctional rhythm was absent or AVNRT remained inducible after initial 20 W ablation, power escalation to ≥25 W was performed. Power escalation was performed at the discretion of the treating physician, guided by persistent inducibility of AVNRT or the absence of slow-accelerated junctional rhythm despite adequate 20 W applications, without a prespecified quantifiable threshold (e.g., a fixed number of applications or cumulative RF duration). Failed ablation was defined as termination of the procedure with persistent inducible AVNRT. All ablations were performed using the conventional right-sided slow pathway approach within the triangle of Koch; no patient underwent a left-sided or non-coronary cusp approach.

The successful slow pathway ablation sites were categorized into three anatomical regions. The posterior region included sites along the tricuspid septal annulus extending to the inferoposterior interatrial septum near the coronary sinus ostium. The anterior region was near the His-bundle catheter along the tricuspid septal annulus. The mid-region was located between the posterior and anterior regions.

### Study outcomes

The primary efficacy endpoints were the acute procedural success and post-ablation recurrence. Acute procedural success was categorized as slow pathway ablation, defined as loss of the AH jump, or slow pathway modification, defined as a persistent AH jump and/or no more than one residual AV nodal reentrant echo beat, in the absence of inducible AVNRT with or without isoproterenol administration. Failed ablation was defined as persistent induction of typical AVNRT at the end of the procedure. The primary safety endpoint was the incidence of serious adverse events, particularly AV block, occurring during the procedure or follow-up. An independent clinical events committee adjudicated procedure-related serious adverse events.

### Follow-up

Routine follow-up visits were scheduled at 1, 6, 12, 18, and 24 months post-procedure. Patients were also instructed to contact the study team if they experienced symptoms of tachycardia or bradycardia.

### Statistical analysis

Continuous variables are presented as mean ± standard deviation and categorical variables as frequencies. Group differences were performed using the *χ*^2^-test for categorical variables and Student's *t*-test for continuous variables. Univariable logistic regression was used to identify predictors of requiring power escalation above 20 W. A multivariable logistic regression model including age, pre-ablation heart rate, and maximum temperature was constructed to assess their independent associations with requiring power escalation. Diabetes mellitus, valvular heart disease, chronic obstructive pulmonary disease, chronic kidney disease, and prior stroke could not be entered into the multivariable model because of zero events in one of the comparison groups (complete separation), which precludes stable parameter estimation for these variables. Odds ratio (OR) with 95% confidence interval (CI) were calculated per 10-year increase for age and per 10-bpm increase for heart rate to provide clinically interpretable effect estimates, and per 1 °C increase for temperature. Missing values were addressed using complete-case analysis. Center-level clustering was not modeled because only three centers participated and only 33 patients required power escalation, limiting reliable estimation of a center-level effect; all centers followed the same study protocol. No formal sample-size or power calculation was performed; the target enrollment of 203 patients was determined based on feasibility during the study period. Given the rarity of permanent AV block (historically 0.1%–0.8%), this study was not powered to formally test for a between-group difference in this safety endpoint, and the findings for this outcome should be considered hypothesis-generating rather than confirmatory. A *p* value < 0.05 was considered statistically significant. Statistical analysis was performed using commercially available software (SPSS 30 for Windows; SPSS Inc., Chicago, IL, USA).

## Results

### Baseline characteristics

The baseline clinical characteristics of the study patients are summarized in Table 1. The mean age of the 203 patients was 54.4 ± 17.3 years, with 82 men and 121 women. Hypertension was present in 68 patients (33.5%), and diabetes in 31 patients (15.3%).

**Table 1:** Clinical characteristics of the study patients.

Number of patients	203
Age (years)	54.4 ± 17.3
Female, *n* (%)	121 (59.6%)
Hypertension	68 (33.5%)
Diabetes	31 (15.3%)
Coronary artery disease	12 (5.9%)
Valvular heart disease	0
Chronic lung disease	0
Stroke	4 (2.0%)

Values are expressed as the mean ± standard deviation or *n* (%).

### Ablation procedures

Procedural data are summarized in Table 2. Initial success at 20 W was achieved in 170 of 203 patients (83.7%). Successful ablation sites were located in the posterior region in 83 cases, the mid-region in 72 cases, and the anterior region of the triangle of Koch (ToK) in 15 cases. The mean number of RF applications required to achieve slow-accelerated junctional rhythm was 6.1 ± 4.8 in patients with initial success at 20 W and 16.8 ± 14.9 in those requiring higher power.

**Table 2:** Study outcomes.

Acute procedural outcomes	
Number of patients	203
Ablation energy	
20 W	170 (83.7%)
Increased to 25 W	17 (8.4%)
Increased to >25 W	16 (7.9%)
Result of ablation	
Slow pathway ablation	87 (42.9%)
Slow pathway modification	115 (56.7%)
Failed ablation	1 (0.5%)
Transient VA block during ablation	48 (23.6%)
Transient AV block (second- or third-degree AV block)	4 (1.9%)
Second degree (Mobitz type I)	3 (1.5%)
Third degree	1 (0.5%)
Long term outcomes	
Permanent AV block (second- or third-degree AV block)	0
Late recurrence of tachycardia	2 (0.9%)

AV, atrioventricular; VA, ventriculoatrial.

Among 33 patients who required higher power escalation beyond 20 W, 17 (8.4%) achieved success with 25 W, and 16 (7.9%) required >25 W. In all 33 patients, ablation was performed in the posterior or mid-region of the ToK. Overall acute procedural success was achieved in 202 of 203 patients (99.5%), with one patient (0.5%) having an unsuccessful procedure. Compared with those who achieved initial success at 20 W, patients requiring higher power were significantly younger (43.8 ± 17.1 vs. 56.4 ± 16.5 years, *P* < 0.001) and had higher pre-ablation heart rates (84.8 ± 11.8 vs. 77.3 ± 10.8 bpm, *P* < 0.001) (Table 3). Univariable logistic regression showed an inverse association between age and the need for power escalation (OR 0.66 per 10-year increase, 95% CI 0.54–0.83; *p* < 0.001), and positive associations with pre-ablation heart rate (OR 1.88 per 10-bpm increase, 95% CI 1.30–2.73; *p* < 0.001) and maximum temperature (OR 1.11 per 1 °C increase, 95% CI 1.02–1.20; *p* = 0.015). On multivariable analysis including age, heart rate, and maximum temperature simultaneously, all three variables remained independently associated with the need for higher power (age: adjusted OR 0.69 per 10-year increase, 95% CI 0.55–0.86, *p* = 0.001; heart rate: adjusted OR 1.67 per 10-bpm increase, 95% CI 1.12–2.48, *p* = 0.011; maximum temperature: adjusted OR 1.10 per 1 °C increase, 95% CI 1.01–1.20, *p* = 0.035). Several comorbidities were absent or rare in the >20 W group, limiting reliable estimation of their effects. (Table 4, Figure 1).

**Table 3:** Comparison between the group with initial success at 20 W and the group requiring higher power.

Clinical characteristics	Initial success at 20 W (*N* = 170)	Requiring higher power (*N* = 33)	*P* Value
Age	56.4 ± 16.5	43.8 ± 17.1	<0.001
Female sex, *n* (%)	103 (60.6%)	18 (54.5%)	0.518
Hypertension	58 (34.1%)	10 (30.3%)	0.671
Diabetes	31 (18.2%)	0	0.003
Valvular heart disease	0	0	N/A
Coronary artery disease	9 (5.3%)	3 (9.1%)	0.397
Chronic lung disease	0	0	N/A
Stroke	4 (2.4%)	0	0.373
Procedural characteristics			
Ablation site			0.060
Ablation at the posterior region of the ToK, *n* (%)	83 (48.8%)	9 (27.3%)	
Ablation at the mid-region of the ToK, *n* (%)	72 (42.4%)	18 (54.5%)	
Ablation at the anterior region of ToK, *n* (%)	15 (8.8%)	6 (18.2%)	
Mean RF applications	6.1 ± 4.8	16.8 ± 14.9	<0.001
Session with obtained slow-accelerated junctional rhythm	4.9 ± 4.6	8.4 ± 6.4	0.002
Procedure duration (min)	82.2 ± 22.0	112.4 ± 30.1	<0.001
Fluoroscopic time (min)	13.2 ± 8.2	21.5 ± 11.2	<0.001
Heart rate before ablation	77.3 ± 10.8	84.8 ± 11.8	<0.001
Total RF delivery time (sec)	221.3 ± 140.8	496 ± 442.3	0.001
ECG characteristics			
Pre-PR interval (ms)	159.8 ± 26.2	151.0 ± 27.8	0.039
Post-PR interval (ms)	160.5 ± 27.2	156.0 ± 23.3	0.325

Values are expressed as the mean ± standard deviation or *n* (%).

AV, atrioventricular; ToK, triangle of Koch; RF, Radiofrequency.

**Table 4:** Univariable predictors of requiring higher power (above 20 W).

Variable	Higher power (*N* = 33)	Initial success at 20 W (*N* = 170)	OR (95% CI)	*P* Value	Adjusted OR (95% CI)	Adjusted *P* Value
Female sex	18/33 (54.5%)	103/170 (60.6%)	0.78 (0.37–1.65)	0.518	—	—
Age (per 10 years)	43.8 ± 17.1	56.4 ± 16.5	0.66 (0.54–0.83)	<0.001	0.69 (0.55–0.86)	0.001
Hypertension	10/33 (30.3%)	58/170 (34.1%)	0.84 (0.37–1.88)	0.671	—	—
Diabetes mellitus	0/33 (0.0%)	31/170 (18.2%)	NE	0.003	—	—
Valvular disease	0/33 (0.0%)	0/170 (0.0%)	NA		—	—
Coronary artery disease	3/33 (9.1%)	9/170 (5.3%)	1.79 (0.46–6.99)	0.403	—	—
Chronic lung disease	0/33 (0.0%)	0/170 (0.0%)	NA		—	—
Chronic kidney disease	0/33 (0.0%)	1/170 (0.6%)	NE	1.000	—	—
Prior stroke	0/33 (0.0%)	4/170 (2.4%)	NE	1.000	—	—
Heart rate before ablation (per 10 bpm)	84.8 ± 11.8	77.3 ± 10.8	1.88 (1.30–2.73)	<0.001	1.67 (1.12–2.48)	0.011
Maximum temperature (per 1 °C increase)	55.8 ± 5.0	53.7 ± 4.2	1.11 (1.02–1.20)	0.015	1.10 (1.01–1.20)	0.035

Categorical variables are presented as n/N (%), and continuous variables as mean ± SD. Odds ratios (ORs) and *p* values were estimated using univariable logistic regression. For variables with complete separation (zero cell counts), ORs are not estimable (NE) and *p* values are from Fisher's exact test. Variables with no variability in the cohort are marked as not applicable (NA). Max temperature had 1 missing value in the success group and was analyzed using complete cases. “Higher Power” and “Initial Success” refer to the group requiring higher power (*N* = 33) and the group with initial success at 20 W (*N* = 170), respectively. Adjusted OR (95% CI) and adjusted *P* value reflect a multivariable logistic regression model including age, heart rate, and maximum temperature simultaneously (*N* = 202); other variables could not be included because of complete separation or absence of variability, as noted above.

**Figure 1:**
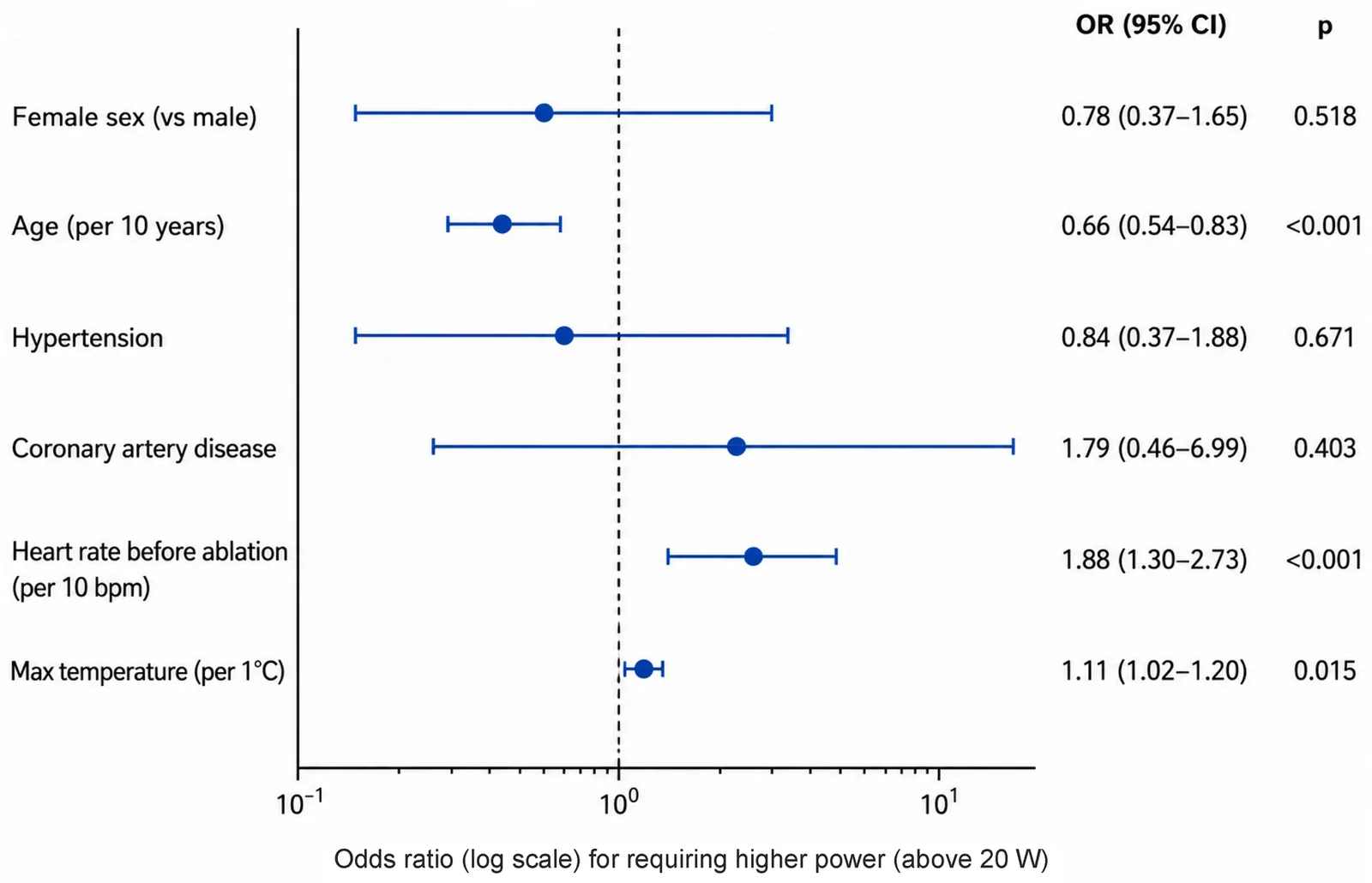
Forest plot of univariable predictors of requiring higher power (above 20 W). The *x*-axis is on a log scale; the dashed vertical line indicates OR = 1. Variables with complete separation or no variability were not plotted: diabetes mellitus (Fisher *p* = 0.003), chronic kidney disease (Fisher *p* = 1.000), prior stroke (Fisher *p* = 1.000), valvular heart disease and chronic lung disease (no variability).

VA block during junctional ectopy occurred in 48 patients (23.6%) during RF application. Transient anterograde AV block was observed in 4 patients (1.9%), including three cases of second-degree Mobitz type I blocks and one case of third-degree AV block. All transient AV blocks occurred either after the acceleration of slow-accelerated junctional rhythm or during VA block assessment, prompting immediate cessation of RF application. All events were resolved completely before the end of the procedure. No patient developed permanent AV block (0/203, 0%; 95% CI, 0%–1.8%), and no patient required pacemaker implantation.

### Follow-up observation

Patients were followed for an average of 18.3 ± 4.1 months. Among those who experienced transient AV block during the procedure, no recurrence of AV block was observed, and none required permanent pacemaker implantation. AVNRT recurred in two patients (0.9%), both of whom underwent repeat ablation with 30 W. No adverse events were reported during follow-up.

## Discussion

The findings of this study support the feasibility of an initial low-power RF strategy with selective power escalation for slow pathway modification in patients with typical AVNRT. In this study, 83.7% of patients achieved successful ablation with an initial power of 20 W. Younger age and higher pre-ablation heart rate were associated with the need for higher power. Over a mean follow-up of 18.3 ± 4.1 months, no patients developed permanent AV block requiring pacemaker implantation, and the recurrence rate following low-power ablation was 0.9%. These results support the feasibility of initiating ablation at 20 W with selective power escalation when necessary.

The risk of permanent AV block extends beyond the immediate post-procedural period and may manifest months or even years after ablation. Previous studies have reported cases of very late-onset second- or third-degree AV block following AVNRT ablation (17, 18). Transient retrograde VA block during junctional rhythm or the development of fast-accelerated junctional rhythm have been identified as predictors of AV block during slow pathway ablation. Accordingly, prompt recognition of these signs and immediate discontinuation of RF delivery are essential to prevent irreversible injury. In this study, 48 patients (23.6%) experienced transient VA block; RF delivery was immediately discontinued, and no progression to permanent AV block was observed.

Several strategies have been proposed to reduce the risk of AV block, including temporarily interrupting RF delivery at the onset of junctional rhythm and performing multiple short applications lasting ≤30 s (19). Liberman et al. (20) suggested rapid atrial pacing during ablation to confirm preserved anterograde AV conduction. However, if junctional rhythm is slightly faster than the pacing cycle length, it may be difficult to recognize, potentially delaying energy cessation. Low-power ablation has also been investigated as a strategy to reduce AV block risk (8, 10, 21, 22). Table 5 summarizes previous findings alongside the results of the present study. Dechering & Schleberger et al. reported similar acute success between high-power (50 W) and moderate-power (30 W) ablation, with a slightly higher recurrence in the 30 W group (21). Langberg et al. demonstrated the efficacy of a stepwise escalation strategy beginning at 10 W and increasing to 30 W, effectively eliminating the slow pathway while avoiding unnecessary high-power applications (10). Bortone et al. also supported a gradual power titration starting from low-energy (8). In the present study, we evaluated an initial low-power ablation strategy (20 W) with selective stepwise escalation. Previous studies indicated that junctional rhythm tends to be unstable at power settings below 20 W, suggesting that 20 W represents the minimum effective threshold (8, 10). Based on these findings, we selected 20 W as the initial power setting. This approach yielded a high acute success rate (99.5%) and a low recurrence rate (0.9%), with no cases of permanent AV block. It should be noted that the studies summarized in Table 5 largely employed fixed power protocols (e.g., a fixed 50 W or 30 W setting), whereas the present study used a step-up strategy beginning at 20 W; consequently, the acute success rate in our cohort includes patients who ultimately required escalation above 20 W. These comparisons should therefore be interpreted as contrasting a low-power-first strategy with fixed moderate- or high-power protocols, rather than as a direct comparison of 20 W vs. 50 W.

**Table 5:** Comparison of studies evaluating energy power for slow pathway ablation.

	Jung & Kwon et al.	Dechering & Schleberger et al. (21)	Langberg et al. (10)	Bortone et al. (8)	Wasmer et al. (4)	Katritsis et al. (22)
Study type	Prospective	Randomized (50 W vs. 30 W)	Randomized (Anterior vs. Posterior)	Retrospective	Retrospective	Prospective
Number	203 (170 in 20 W, 33 in above 20 W)	634 (342 in 50 W vs. 292 in 30 W)	50 (28 vs. 22)	468	2,101	1,087
Energy power (Watt)	20 W (Above 20 W: 16.3%)	50 W vs. 30 W	10 W to 30 W	From 5 W, Mean 31.7 ± 3.0	N/A	20 W to 40 W
Mean number of RF applications	6.1 ± 4.8 vs. 16.8 ± 14.9	5.9 ± 6.6 vs. 7.6 ± 8.5	9 ± 5 vs. 7 ± 5	3.2 ± 1.1	5 ± 7	N/A
Acute success (%)	99.5	98.8 vs. 97.7	96	98.7	98	N/A
FU duration (months)	18.3 ± 4.1	39 ± 7.5 vs. 31 ± 11.4	N/A	28.1 ± 14.1	62 ± 43	3
Redo ablation or recurrence (%)	0.9	4.6 vs. 6.8	N/A	3.6		2.2
Transient AV block (%)	1.9	2.6 vs. 1.7	N/A	1.9	2	0.1
Permanent AV block (%)	0	0 vs. 0	2	0.2	0.2	0.1

Transient anterograde AV block during ablation not only necessitates immediate interruption of RF delivery but may also result in serious complications requiring pacemaker implantation. Higher ablation energy and target temperature levels are expected to increase the likelihood of AV block or VA block during junctional ectopy (12). The potential relationship between lower RF power and the risk of irreversible AV nodal injury warrants further investigation in controlled comparative studies. In this study, transient AV block was rare (1.9%), consisting predominantly of second-degree AV block (Mobitz type I), all resolving within 30 s.

On multivariable analysis, age, heart rate, and maximum temperature remained independently associated with the need for higher power. Younger age and higher pre-ablation heart rate were the principal baseline factors associated with power escalation. Potential explanations include greater convective cooling at higher heart rates, reduced catheter stability with rapid cardiac motion, insufficient lesion formation at 20 W in some patients, and possible age-related differences in slow pathway anatomy or tissue characteristics (11). The association between higher maximum temperature and the need for power escalation remains unclear but may reflect procedural factors related to catheter position or catheter–tissue interaction.

From our clinical perspective, the overall acute success rate of 99.5% achieved with an initial low-power strategy appears acceptable, particularly given the absence of permanent AV block. Although Bortone et al. previously demonstrated the utility of a low-power strategy, their method—incrementing power by 5 W every 5 s up to 10 W above the threshold for junctional rhythm and maintaining energy delivery for 120 s—may be challenging to implement in routine practice (8, 21). In contrast, our strategy is simple, reproducible, and requires no additional procedural steps beyond selecting a low initial power and considering the baseline heart rate before energy delivery.

### Study limitations

This study has several limitations. First, it is a relatively small-scale, non-randomized prospective study. Although all consenting patients with typical AVNRT were enrolled during the study period, selection bias may still be present. Additionally, the small sample size may have limited statistical power to detect complications or recurrence. Given that the incidence of permanent AV block typically ranges from 0.1% to 0.8%, the sample size of 203 patients may be underpowered to assess this rare outcome, although the absence of permanent AV block in our cohort is consistent with the low incidence reported in previous studies (2–4). Therefore, the absence of permanent AV block in the present cohort should be interpreted descriptively and does not establish a reduction in risk compared with conventional higher-power ablation. In addition, this study was a single-arm cohort in which all patients received the initial 20 W strategy, without a concurrent comparator group treated with a standard- or higher-power protocol at the same centers during the same enrollment period; the comparisons summarized in Table 5 are drawn from separate historical studies with different patient populations, eras, and catheter technologies. Therefore, this study cannot determine the precise energy threshold required for effective and safe slow pathway ablation, and the findings should be interpreted as hypothesis-generating rather than confirmatory. Second, the optimal energy level may vary depending on catheter-tissue contact. In this study, all operators made efforts to ensure adequate catheter-tissue contact to achieve successful ablation at low-power. However, since a contact force-sensing catheter was not used, variability among operators may exist. As a result, the required energy may differ depending on the degree of contact, and procedural outcomes may be influenced by the operator's experience and expertise. In addition, because power escalation to ≥25 W was performed at the discretion of the treating physician rather than according to a standardized, quantifiable threshold (e.g., a fixed number of applications or cumulative RF duration), operator-dependent variability in the decision to escalate power cannot be excluded. Third, a three-dimensional electroanatomical mapping system was not used in this study. This was because the study was limited to first-time AVNRT ablation cases, in which a three-dimensional mapping system—and consequently contact force–sensing catheters—is not reimbursed under the Korean National Health Insurance system. Therefore, all procedures in this study were performed using a conventional fluoroscopy-guided approach. As a result, precise measurement of the spatial distance between the ablation site and the His bundle was not feasible, which should be considered when interpreting the procedural findings. All procedures were performed by experienced electrophysiologists who each performed more than 50 AVNRT ablation procedures annually and had performed more than 500 AVNRT ablation procedures in total, and an impedance drop of ≥10% was used as a surrogate marker of adequate catheter-tissue contact during ablation. Fourth, because all ablation catheters used in this study were non-irrigated tip catheters, these findings may not be directly applicable to procedures performed with irrigated-tip catheters. Furthermore, although three different catheter types were used according to operator preference, catheter type, operator, and treatment site were not formally examined as covariates for power escalation, which should be considered a further limitation of this study. Center-level clustering was also not formally modeled because of the small number of participating centers and power-escalation events. Finally, the mean follow-up duration of 18.3 ± 4.1 months is sufficient to assess early outcomes but may be inadequate to capture very late recurrences of AVNRT. Moreover, the relatively short follow-up period limits the ability to detect late-onset AV block.

## Conclusion

An initial low-power (20 W) strategy with selective power escalation achieved a high acute procedural success rate, and no permanent AV block was observed in this prospective multicenter cohort. These findings support the feasibility of a low-power-first approach for typical AVNRT, although prospective comparison with a concurrent standard- or high-power arm is needed to determine its relative safety and efficacy.

## Data Availability

The raw data supporting the conclusions of this article will be made available by the authors, without undue reservation.
